# The Continuous Incorporation of Carbon into Existing *Sassafras albidum* Fine Roots and Its Implications for Estimating Root Turnover

**DOI:** 10.1371/journal.pone.0095321

**Published:** 2014-05-02

**Authors:** Thomas S. Adams, David M. Eissenstat

**Affiliations:** Department of Ecosystem Science and Management and the Ecology Graduate Program, the Pennsylvania State University, University Park, Pennsylvania, United States of America; University of Nottingham, United States of America

## Abstract

Although understanding the timing of the deposition of recent photosynthate into fine roots is critical for determining root lifespan and turnover using isotopic techniques, few studies have directly examined the deposition and subsequent age of root carbon. To gain a better understanding of the timing of the deposition of root carbon, we labeled four individual *Sassafras albidum* trees with 99% ^13^C CO_2_. We then tracked whether the label appeared in roots that were at least two weeks old and no longer elongating, at the time of labeling. We found that not only were the non-structural carbon pools (soluble sugars and starch) of existing first-order tree roots incorporating carbon from current photosynthate, but so were the structural components of the roots, even in roots that were more than one year old at the time of labeling.Our findings imply that carbon used in root structural and nonstructural pools is not derived solely from photosynthate at root initiation and have implications regarding the determination of root age and turnover using isotopic techniques.

## Introduction

The timing of the deposition of photosynthetically derived carbon into roots is of considerable scientific importance because it has implications both for factors controlling root physiology and lifespan and for the determination of root age and turnover using isotopic techniques. Despite this importance, and over two decades of work on the subject, there is still no clear consensus concerning the timing of the deposition of root carbon. Until recently, it has been assumed that both root nonstructural storage carbon and structural carbon are primarily deposited when a root is first initiated. Marshall and Waring [1] contended that nonstructural root storage carbohydrates, such as starch, are deposited solely when the root is first formed and it is the rate of depletion of these reserves that determines root longevity. Recent attempts to determine root age and root turnover using isotopic techniques, both bomb ^14^C and ^13^C labeling, have assumed that root structural tissue originates within the year that a root is formed and that no new carbon is subsequently added to the root [2]–[8]. However, to our knowledge, these assumptions concerning the timing of the origin of root structural and nonstructural carbon have not been adequately tested.

A number of studies have called into question the validity of the assertion by Marshal and Waring [1] that root nonstructural carbohydrate reserves (e.g., starch) are solely created when a root is first born. For example, Nguyen *et al*. [9] found that root carbohydrate levels increased late in the growing season, implying that root carbohydrate reserves were not solely laid down when a root is first formed. Additionally Kosola *et al*. [10] found in a study that tracked individual roots, that the root starch concentration of Eugenei hybrid poplars did not decline with root age. As a result, they concluded that the starch reserves in the fine roots of the poplar were not determined at root birth and that these reserves were “labile and dynamic”.

Structural carbon has also traditionally been viewed as originating from recent photosynthate during root formation [2]–[8]. But recent studies using bomb ^14^C have found that the origin, and subsequent age, of root structural carbon may involve more complicated processes than previously assumed. Evidence now suggests that stored carbon fixed prior to root formation may be incorporated in the structural tissue of newly formed roots [3], [6], [8], [11]. Furthermore, the amount of stored carbon incorporated in the formation of larger diameter fine roots (1.5–2 mm), presumably of higher root order [12], is higher than in smaller diameter, lower order roots (<0.5 mm) [6]. Because root age is positively correlated with root diameter [13]–[15], it is difficult to disentangle the relationship between root age assessed by direct observation, root diameter, and estimated age based on carbon residence time.

In an attempt to gain a better understanding of the timing of the deposition of root carbon, we labeled *Sassafras albidum* trees with 99% ^13^CO_2_. We then tracked whether the label appeared in first-order roots that were at least two weeks old and no longer elongating at the time of labeling. Based on previous studies, we hypothesized that the ^13^C label would appear in the nonstructural carbon pools of the existing first-order roots, but not in their structural carbon pool.

## Materials and Methods

**Figure 1:**
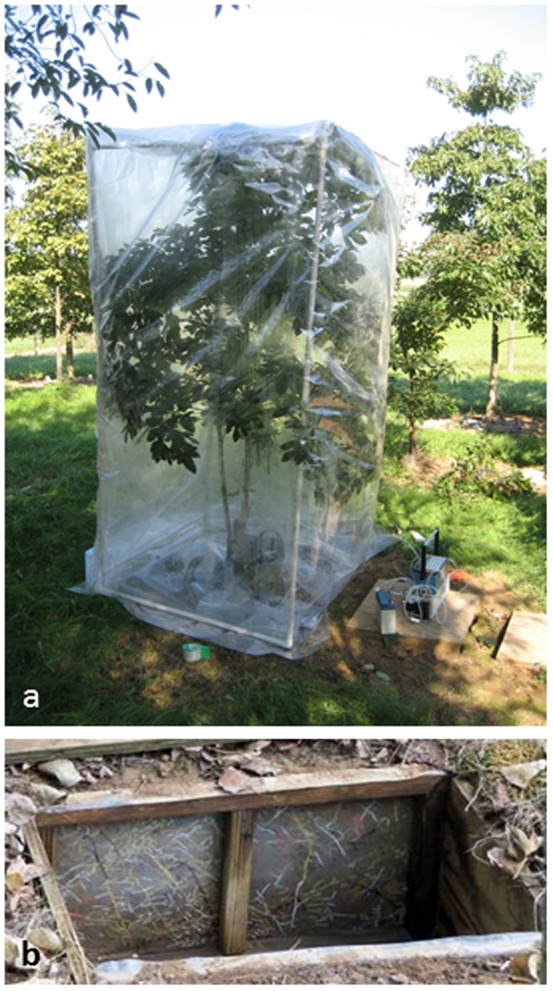
Methods used in study. **A**. *Sassafras albidum* tree covered in a clear mylar balloon during the ^13^CO_2_ labeling process. **B**. Example of a root box showing roots that were traced on a clear acetate window facing the study tree using different color paint pens.

This study was conducted at a common garden planting (mixed species planting) located at the Russell E. Larson Agricultural Research Center, Pennsylvania State University, USA (40.8°N, 77.9°W) [16]. For this study we labeled *Sassafras albidum* with 99% ^13^CO_2_ (Cambridge Isotope Laboratories, Andover, MA USA) (Figure 1). We chose this species because of its comparatively small size (ca. 2.5 m tall) relative to other tree species in the garden, and the easily recognizable, relatively large diameter, fine roots which emit a distinctive odor when harvested thereby precluding confusion with the roots of neighboring trees. Each species plot represented six trees planted in a double row with spacing of 3 m between trees within the row, 3 m between the double rows and 5 m between the six-tree plots.

Soils at the common garden are relatively fertile Hagerstown silt loam, well-drained, with a pH ranging from 6.1–6.5 and with some areas high in calcium. Previous to planting the trees, the site was used as a grass hayfield. The entire area was fenced to keep out deer. *Sassafras albidum* trees were collected in the early spring of 1996 from seedlings around State College, PA and therefore were approximately 13 years old at the time of the experiment. Understory vegetation was controlled within a half-meter of the tree using weed barrier cloth and gravel mulch and sprayed with glyphosate to a distance of about two meters from the trunk. Further from the trees, grass was mowed weekly or longer, depending on climatic conditions.

Root boxes were installed in the spring of 2008 and again in the spring of 2009[17]. Briefly, a 0.6×0.5×0.4 m hole was dug in the ground approximately 0.5 m in front of each study tree. A 0.6×0.5×0.4 m box constructed of 1.9 cm treated plywood, with a clear 0.127 mm thick (5-mil) acetate window, was then placed in the ground with the acetate window facing the tree (Figure 1B). A removable piece of 1.3 cm thick foam insulation board was placed against the window when not in use to minimize temperature differences between the soil and the interior of the root box. In this fashion, roots growing against the acetate window could be followed from birth by tracing the roots on the acetate using different colored paint pens (Marvy DecoColor pens, Uchida of America Corp., Torrance, CA, USA) on different tracing dates. Three small first-order roots per tree were harvested prior to labeling and pooled into a single pre-label sample per tree to assess background ^13^C levels. After labeling the trees with ^13^C, preexisting roots of a known age were then excised by cutting through the acetate window and individual first-order roots were analyzed for ^13^C in structural and nonstructural carbon pools.

*Sassafras albidum* trees, approximately 2.5 m in height, were labeled with 99% ^13^CO_2_ by first surrounding the trees in a 2.4×1.2×1.2 m clear mylar balloon supported by a frame constructed of 1.9 cm internal diameter pvc pipe (Figure 1A). Mylar was also used to cover the soil surface to minimize non-target ^13^CO_2_ uptake. During the labeling, air was constantly circulated in the balloon using three small battery powered fans hung within the canopy of the tree. Carbon dioxide concentrations within the balloon were measure during the labeling using a Li-Cor 6200 (Li-Cor Biosciences, Lincoln, NE, USA). The labeling itself occurred by releasing a short pulse of 99% ^13^CO_2_ into the balloon and waiting until the CO_2_ concentration in the balloon dropped back below background levels (∼385 ppm). This process took approximately 30 minutes per tree once the balloon was installed. Roots were collected immediately prior to labeling and at various times after labeling. Only first-order, distal, roots were sampled from the root boxes for ^13^C analysis. All roots sampled from the trees were at least two weeks old at the time of labeling and were no longer actively elongating. Three trees were labeled on August 5^th^, 2008 and based on these results a fourth tree was labeled the following year on September 3^rd^, 2009 to enable the sampling of roots of greater age at the time of labeling as well as to examine in greater detail the fate of the label in both the structural and the non-structural root carbon pools. Only a single tree was labeled in 2009 due to a lack of additional suitably sized trees.

**Figure 2:**
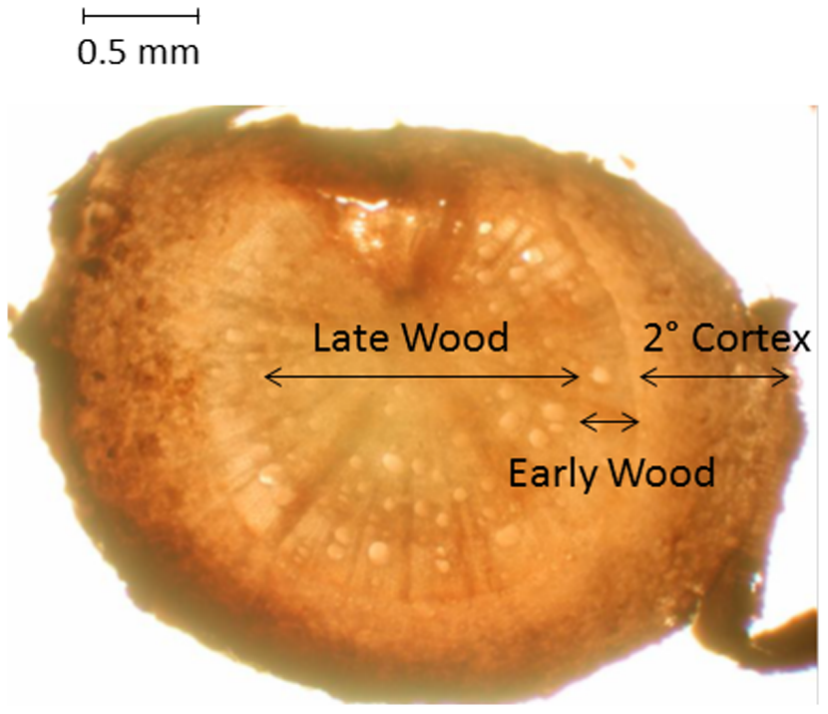
*Sassafras albidum* root cross-section. Micrograph depicting how higher-order roots (approximately 4^th^ or 5^th^ order) were dissected prior to δ^13^C analysis in 2008.

In 2008 all roots visible through the acetate windows were traced 19 days (d) prior to labeling for each of three trees labeled with 99% ^13^CO_2_ and an unlabeled control tree. The unlabeled control tree was used to insure accurate background ^13^C measurements. Root samples were excised from the root boxes immediately prior to labeling and three times after labeling (3, 6 and 19d). Additional larger diameter, higher-order roots (approximately 4^th^ or 5^th^ order) were dug from the soil directly beneath the three labeled trees and the unlabeled-control tree four days after labeling. The upper, non-distal, portion of two roots from each of these samples were then cut into segments and dissected under the microscope into three anatomical categories: late wood, early wood, and secondary cortex (Figure 2); where the late wood accounts for the inner most portion of the root cross section, surrounded by the early wood and finally the secondary cortex. Because these roots were not followed in the root boxes, their precise age was unknown, but based on the development of early and late wood; they were at least one year old at the time of labeling [18]. Individual sampled roots were freeze dried, ground, and then underwent acid-base-acid washing until the supernatant was clear to remove nonstructural carbon [4]. The remaining sample was oven dried at 60°C for 24 hrs, weighed, and placed into tin capsules.

In 2009, a single Sassafras tree was labeled with ^13^CO_2_. Only one tree was labeled in 2009 and less ^13^CO_2_ was used than in 2008 due to a decreased number suitable unlabeled trees. Because there was no statistical difference (*P* = 0.40) in isotopic signature between the roots of the control tree and the roots sampled prior to labeling the labeled trees in 2008, we did not sample the roots of a second control tree in 2009. Roots were traced weekly prior to labeling, with three root flushes observed at 71d, 28d, and 16d prior to labeling. The weekly tracing interval did not allow for the determination of exact root ages in each flush, as roots could have been born on any of the previous seven days. For analysis, we assigned root birth as the day of initial observation, so a root might actually be as much as 7d older than the assigned birth date. Individual first-order roots were sampled immediately prior to labeling and twice after labeling, once at five days after labeling and once at 14 days after labeling. These individual sampled first-order roots were ground and then underwent a stepwise process to isolate soluble sugars, starch and structural carbon. This involved first boiling the root samples in Millipore water (Millipore, Billerica, MA USA) and sampling the resulting supernatant for soluble sugar ^13^C content. The remaining solid sample was then re-suspended in Millipore water and digested with 0.5 M sodium acetate and 5 units of amyloglucosidase and 2.5 units alpha amylase [19]. The resulting supernatant was then analyzed for starch ^13^C content. Finally, the remaining root sample underwent the acid-base-acid cleaning mentioned above, leaving structural carbon for ^13^C analysis. Solid samples were oven dried at 60°C for 24 hrs, weighed, and placed in tin capsules. Liquid samples were pipetted into pre-weighed tin capsules, oven dried at 60°C for 24 hrs, and then re-weighed.

All samples from both years of the study were analyzed for ^13^C content at the UC Davis Stable Isotope Facility. Statistical analyses were conducted using SAS JMP 9.02 (SAS Institute Inc., Cary, NC, USA). Results from each year were analyzed using an ANOVA with sampling date, tree, and labeling treatment as factors in 2008 and sampling date, root age, root carbon pool, and labeling treatment as factors in 2009, where only one tree was labeled. Non-significant factors were removed from the model. Results from the final model were considered statistically significant at *P*≤0.05 using a one tailed T-test.

## Results

**Figure 3:**
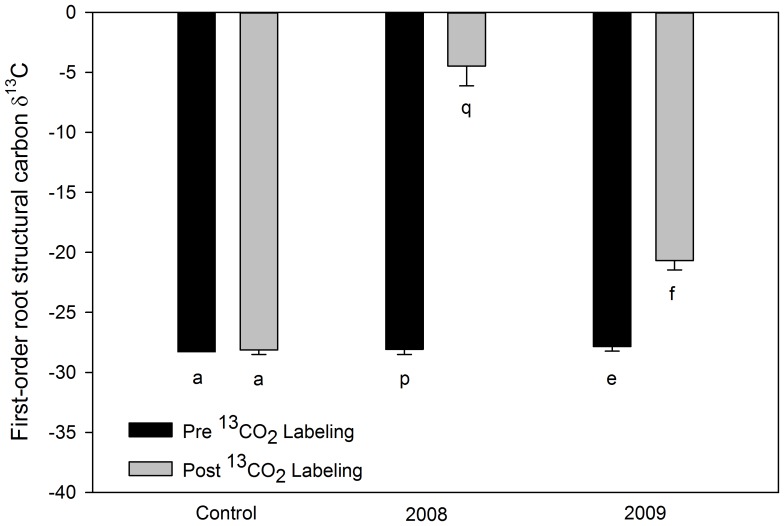
Mean *Sassafras albidum* first-order root structural carbon δ^13^C values. Data from an unlabeled control tree, and trees labeled in 2008 and in 2009. All roots were at least 2 weeks old at the time of labeling and subsequent sampling and some were older than 71 days. Black bars represent roots sampled prior to whole-tree labeling and grey bars represent roots sampled after whole-tree labeling. Note that less negative values indicate greater isotopic enrichment. Error bars denote standard error between individual sampled roots. Different lower case letters denote significant differences at *P*≤0.05 using a one tailed T-test between pre- and post-labeled roots.

**Figure 4:**
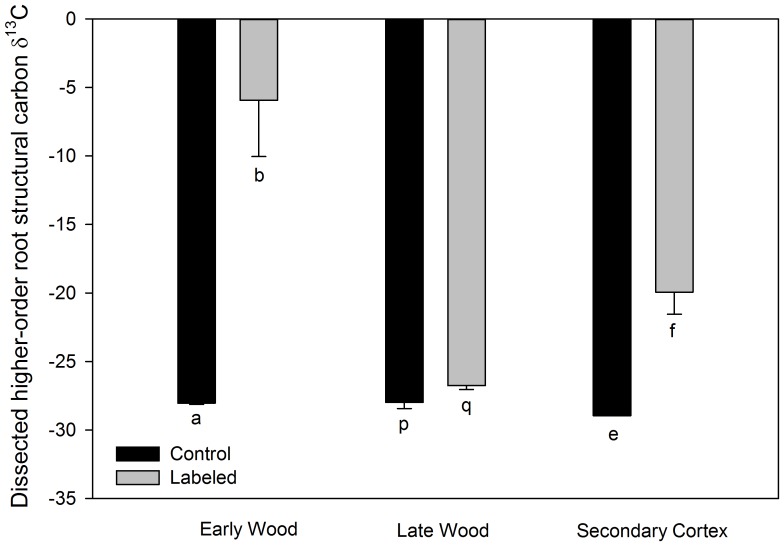
Mean *Sassafras albidum* root structural carbon δ^13^C values from higher-order dissected roots. Trees were labeled and sampled in 2008. Control roots were sampled from unlabeled control trees and labeled roots were sampled from trees labeled with 99% ^13^CO_2_. Late wood refers to the innermost portion of the dissected roots (see Figure 2). Early wood refers to the root tissue immediately surrounding the late wood. Secondary cortex refers to the outermost portion of the root surrounding the early wood. Note that less negative values indicate greater isotopic enrichment. Error bars denote standard error. Different lower case letters denote significant differences at *P*≤0.05 using a one tailed T-test between dissected root tissue from unlabeled control trees and labeled trees.

**Figure 5:**
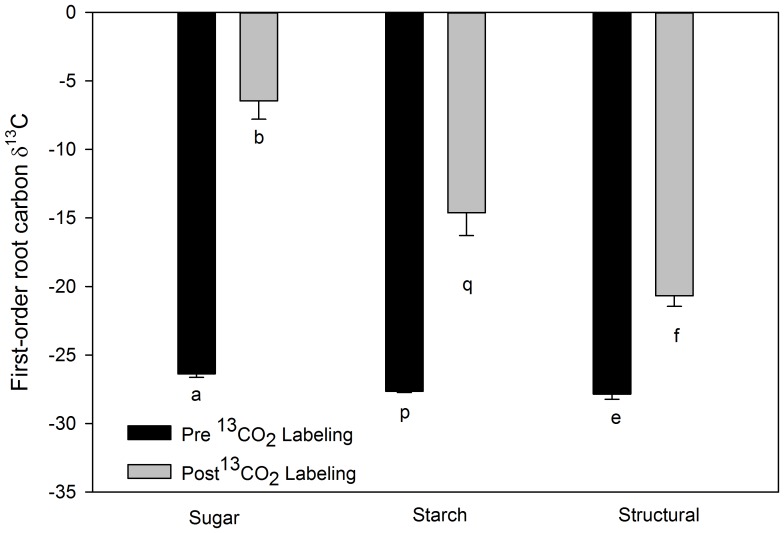
Mean *Sassafras albidum* first-order root carbon δ^13^C values. Roots were at least two weeks old at the time of whole tree labeling in 2009. Black bars represent roots that were sampled immediately prior to whole-tree labeling. Light grey bars represent roots sampled after whole-tree labeling. Note that less negative values indicate greater isotopic enrichment. Error bars denote standard error. Different lower case letters denote significant differences at *P*≤0.05 using a one tailed T-test between pre- and post-labeled roots of each tissue type.

In both 2008 and 2009 structural carbon of the roots of the labeled *S. albidum* trees, which were at least two weeks old at the time of labeling, were significantly (2008 *n* = 35, *P*<0.0001; 2009 *n* = 38, *P* = 0.02) enriched in ^13^C compared with roots sampled from the same trees prior to 13CO_2_ labeling (Figure 3, Table 1). The lower enrichment observed in 2009 likely resulted from less ^13^CO_2_ being used in the labeling process. In 2008, no significant differences were observed among the three labeled trees in the ^13^C enrichment of the roots (*P* = 0.44) nor in timing of the root sampling post labeling (*P* = 0.19). Additionally, in 2008 all portions of the dissected roots (early wood (*ew*), late wood (*lw*), and secondary cortex (*sc*); were significantly enriched in ^13^C by labeling with 99% ^13^CO_2_ (Figure 4) (*ew*: *P* = 0.01, *sc*: *P* = 0.01, *lw*: *P* = 0.04). In 2009, in addition to root structural carbon, both root soluble sugars and root starch were also significantly enriched with ^13^C by labeling (*P* = 0.001 and *P*<0.0001) (Figure 5), with soluble sugars being most enriched followed by starch and then structural carbon. The timing of the root sampling post labeling significantly affected the ^13^C enrichment of the root sugars (*P* = 0.005), with roots sampled later showing decreased ^13^C enrichment. Root age at the time of labeling did not significantly affect ^13^C enrichment of the root sugars (*P* = 0.09). Neither of these factors significantly affected the ^13^C enrichment of the structural carbon pool (root age *P* = 0.15, sample date *P* = 0.14), nor of the starch pool (root age *P* = 0.26, sample date *P* = 0.26) (Table 1).

**Table 1:** *Sassafras albidum* δ*^13^*C root data from trees labeled with 99% ^13^CO_2_.

Year	Root age (d)	Number of days post-labeling		
		3	6	19
*Structural* δ^13^C (*Control* = −28.13±0.24 (*n* = 9))				
**2008**	**>14**	−2.18±3.38 (*n* = 14)	−5.75±1.28 (*n* = 13)	−6.23±3.89 (*n* = 8)
**2009**		**5**	**14**	
*Structural* δ^13^C (*Control* = −27.85±0.38 (*n* = 2))				
	**16**	−16.84±3.73 (*n* = 3)	−18.93±3.76 (*n* = 2)	
	**28**	−19.99±1.46 (*n* = 13)	−21.87±1.22 (*n* = 13)	
	**71**	−21.99±1.91 (*n* = 4)	−22.04±1.87 (*n* = 3)	
*Starch* δ^13^C (*Control* = −27.64±0.09 (*n* = 2))				
	**16**	−16.66±3.91 (*n* = 3)	−12.17±8.08 (*n* = 2)	
	**28**	−14.90±2.57 (*n* = 13)	−15.50±2.70 (*n* = 13)	
	**71**	−17.38±4.51 (*n* = 4)	−5.41±12.77 (*n* = 3)	
*Sugar* δ^13^C (*Control* = −26.38±0.25 (*n* = 2))				
	**16**	−0.68±5.81(*n* = 3)	−9.11±1.98 (*n* = 2)	
	**28**	−0.18±1.37 (*n* = 13)	−10.86±1.77 (*n* = 13)	
	**71**	−2.93±9.11 (*n* = 4)	−14.91±1.04 (*n* = 3)	

Data showing the number of samples (*n*) analyzed as well as the average δ^13^C and associated standard error for each root age class, sampling date, and carbon pool from trees labeled with 99% ^13^CO_2_ in 2008 (n = 3) and 2009 (n = 1), where less negative values indicate greater isotopic enrichment. ^13^CO_2_ labeling significantly enriched the root structural δ^13^C in 2008 (*n* = 35, *P*<0.0001) and 2009 (*n* = 38, *P* = 0.02). Additionally, both the root starch and root sugars were significantly enriched in 2009 (*n* = 38, *P_starch_*<0.0001, *P_sugars_* = 0.001). The number of days sampling occurred post labeling did not significantly affect enrichment in root structural δ^13^C in 2008 (*P* = 0.19) nor root structural δ^13^C nor root starch δ^13^C in 2009 (*P_structural_* = 0.14, *P_starch_* = 0.26). In 2009 root sugars were significantly affected by the days post labeling (*P* = 0.005), with roots sampled later showing decreased enrichment. The age of the roots at the time of labeling did not significantly affect enrichment in any of the carbon pools in 2009 (*P_structural_* = 0.15, *P_starch_* = 0.26, *P_sugars_* = 0.09).

## Discussion

Our results clearly demonstrate that the timing of the deposition of photosynthetically derived carbon into roots involves more complicated processes than has previously been assumed. Not only were the non-structural carbon pools (soluble sugars and starch) of existing first-order tree roots incorporating carbon from current photosynthate (Figure 5), but so were the structural components of the roots (Figures 3 and 4). Our results are in direct contradiction with the assertion of Marshall and Waring [1] that root carbohydrates associated with starch formation are derived only at root initiation. Additionally, the incorporation of current photosynthate into the structural carbon of roots which were at least two weeks old, and in some roots more than a year old, at the time of labeling has implications regarding the determination of root age and turnover using isotopic techniques. For example, when using bomb ^14^C analyses, if the incorporation of new carbon into the structure of existing roots continues to occur over sufficient time scales, an underestimation of the actual age of roots will result since the ^14^C signature of the newly incorporated carbon would be depleted in ^14^C compared to the carbon used to construct the root at initiation. Furthermore, in recent studies that have used the incorporation of ^13^C from current photosynthesis into the structural carbon pools of labeled tree roots to assess root turnover [2], [5], if new structural carbon is continually added to existing roots, root turnover will appear to occur more quickly since the depletion in the ^13^C signal will not be solely derived from the death of labeled roots and the initiation of new root growth.

**Figure 6:**
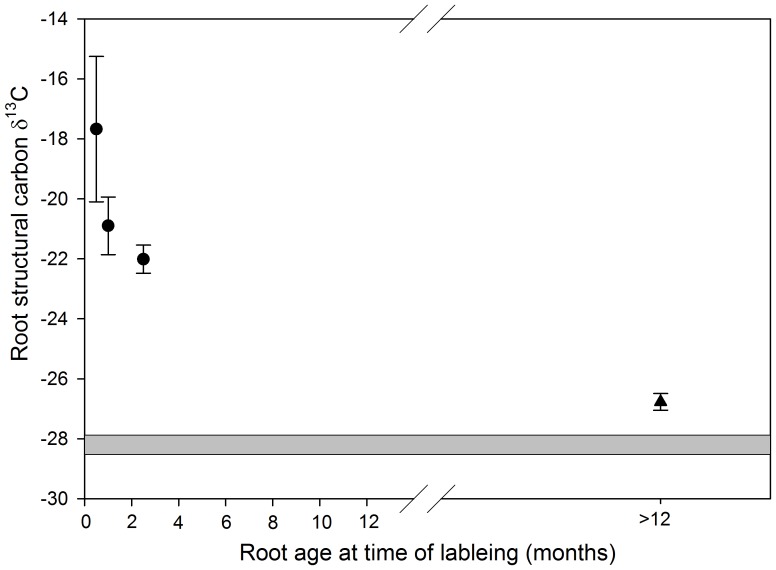
Mean *Sassafras albidum* root structural carbon δ^13^C values verses root age at time of sampling. Circles represent roots sampled in 2009. The triangle represents the mean of the late wood (lw) structural root tissue from the 2008 dissection samples which were older than one year when sampled but the exact age is unknown. The grey horizontal bar shows the 95 percentiles for background δ^13^C structural carbon values from 2008 and 2009. Note that less negative values indicate greater isotopic enrichment. Error bars denote standard error.

Our results demonstrate that current photosynthate is incorporated into the structural tissue of higher-order roots (Figure 4) as well as first-order roots (Figures 3, 5). We also found evidence, when examining the full range of root ages sampled (16d to >1year), that this incorporation appears to decrease with root age (Figure 6). However, even the roots that were older than one year at the time of labeling never were depleted to the level of the non–labeled roots (Figure 6). Additionally, this trend of decreasing incorporation of C in structural tissues with age was not significant when just examining first-order roots sampled within a 2.5-month period.

Because roots of higher branching order must in general be older than the lower order roots they support, the diminished incorporation of current photosynthate with root age over longer time scales suggests that higher-order, longer-lived roots have fewer errors associated with estimates of root lifespans and turnover based on isotopic techniques. However, because roots of higher branching order, unlike lower-order roots, often undergo secondary development, the potential for newly incorporated carbon to bias estimates of root lifespan and turnover may actually increase with increasing root order. One possible way to overcome this bias, based on our observation of decreased recent carbon assimilation in the older tissue of higher-order roots (Figure 4: late wood vs. early wood), would be to use the oldest, inner most root tissue of higher-order woody roots for isotopic analysis.

Although we did not specifically examine the physiological processes involved in the observed incorporation of recent photosynthate into the structural pools of existing roots, secondary cellular processes such as cell wall thickening could be responsible. In another tree species, first-order *Liriodendron tulipifera* roots of known age collected from the same common garden during the same time period showed significant secondary wall thickening between birth and 14 days in the hypodermal and endodermal cells of the primary tissue (Zadworny *et al*. unpublished data). Similar results have been observed among citrus rootstocks when comparing the primary tissue of first- with second-order roots [20]. Regardless of the exact physiological mechanism involved, we have clearly demonstrated that new carbon is incorporated into the carbon pools of existing first-order roots for months after birth and in higher order roots, for potentially years. Because this study involved only one species, further study is needed to determine if the observed assimilation of new carbon into existing non-elongating first-order roots is a broadly occurring process. Additionally, more work is needed to uncover the underlying mechanisms driving the observed assimilation as well as the exact extent to which new carbon may influence estimates of root age and turnover using isotopic techniques.
